# The antibiotic food-chain gang.

**DOI:** 10.3201/eid0703.010337

**Published:** 2001

**Authors:** P Courvalin


of serious gram-positive infections in humans (with no animal use counterparts) are in the pharmaceutical pipeline (e.g., LY333328 and daptomycin) or have recently been approved (e.g., linezolid). We hope that a fair balance can be achieved by the human medical and the animal health and production communities with regard to the types of antimicrobial agents that can be used in each sector.

**Thomas R. Shryock**
Elanco Animal Health, Greenfield, Indiana, USA

### References

1. Courvalin P. Will avilamycin convert ziracine into zerocine? Emerg Infect Dis 2000;6:558.
2. Aarestrup FM. Association between decreased susceptibility to a new antibiotic for treatment of human disease, everninomicin (SCH 27899), and resistance to an antibiotic used for growth promotion in animals, avilamycin. Microb Drug Resist 1998;4:137-41.
3. Schouten MA, Voss A, Hoogkamp-Korstanje JAA, and the European VRE Study Group. Antimicrobial susceptibility patterns of enterococci causing infections in Europe. Antimicrob Agents Chemother 1999;43:2542-6.
4. Jones RN, Hare RS, Sabatelli FJ, Ziracin Susceptibility Testing Group. In vitro gram-positive antimicrobial activity of evernimicin (SCH 27899), a novel oligosaccharide, compared with other antimicrobials: a multicentre international trial. J Antimicrob Chemother 2001;47:15-25.

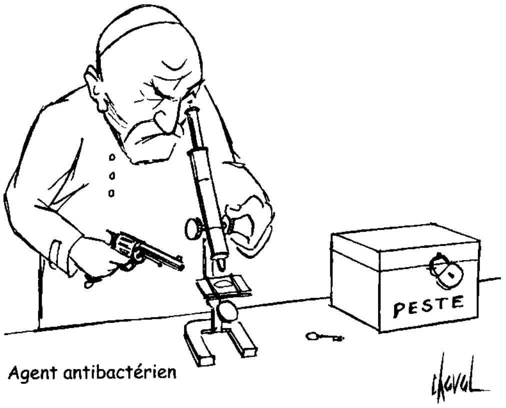

Cartoon from poster of annual meeting of Société Française de Microbiologie, Section des Agents Antimicrobiens. Used with permission and courtesy of P. Courvalin.

### The Antibiotic Food-Chain Gang

**To the Editor:** In his reply to my letter (1), Dr. Shryock states that use of the growth promoter avilamycin, which confers cross-resistance to other members of the everninomycin class of drugs, was in compliance with the Swann principles. The Swann report, issued in 1969, recommends that antibiotics used to treat infections in humans not be used as animal-food additives (2). The combined efforts of many scientists were needed to bring about the 1999 ban in Europe of spiramycin, tylosin, virginiamycin, and bacitracin, each of which confers resistance to antibiotics used in clinical settings. It appears that more than 30 years was necessary for the animal-food industry to act in accordance with the Swann report.

The reasoning in terms of drug structures can be misleading. The implication is that drugs that are chemically closely related have the same target of action and are therefore subject to cross-resistance, and vice versa. For example, because it has an unusual structure, apramycin (a 4-substituted-2-deoxystreptamin) was used exclusively in animals in the hope that it would not be recognized by any of the known aminoglycoside-modifying enzymes (3). However, enterobacteria of animal origin were resistant to apramycin by synthesis of a plasmid-mediated 3-*N*-aminoglycoside acetyltransferase type IV, which also confers resistance to gentamicin (4). Following spread in animal strains (5), the plasmid was later found in clinical isolates from hospitalized patients (6).

The use of antibiotics in general should be based on the mechanisms of resistance in bacteria, rather than on their chemical makeup. In particular, the concept that resistance was a class phenomenon rapidly lost favor because of the extension of the concept of cross-resistance and the increased occurrence of co-resistance.

In classical cross-resistance, a single biochemical mechanism confers resistance to a single class of drugs: use of a given antibiotic can select resistance to other members of the group but not to drugs belonging to other classes. However, cross-resistance between drug classes can occur by two mechanisms: overlapping targets and drug efflux. An example of target overlap is provided by the macrolides, lincosamides, and streptogramins (MLS), which are chemically distantly related. However, constitutive methylation of a single adenine residue in ribosomal RNA confers high-level resistance to the three classes of antibiotics. This resistance phenotype is due to the fact that all these antibiotics have overlapping targets on the ribosome (7). Active efflux of the drugs outside bacteria has recently been recognized as a common resistance mechanism (8,9). This energy-dependent export confers low-level resistance to a wide variety of antibiotics. The broad substrate specificities of the pumps account for decreased susceptibility to beta-lactams, aminoglycosides, tetracyclines, chloramphenicol, trimethoprim, sulfonamides, fluoroquinolones, and MLS, among others (9).

In contrast to cross-resistance, co-resistance is due to the presence in the same host of several mechanisms, each conferring resistance to a given class of drugs. In addition, the corresponding genes are often adjacent (physically linked) and expressed in a coordinated fashion. One of the most efficient system of this type is represented by the integrons (10) first described in gram-negative bacilli (11,12) and more recently found in gram-positive bacteria (13). Because of the genetic organization resulting in co-expression of the various genes, use of any antibiotic that is a substrate for one of the resistance mechanism will co-select for resistance to the others and thus for maintenance of the entire gene set. Since cross-resistance means cross-selection and co-resistance implies co-selection, the use of any antimicrobial agent is de facto rendered inadequate as a growth promoter.

I also disagree with the notion that because a member of an antibiotic class has been misused as a growth promoter the class should not be used in the future for human therapy; the hierarchy could conceivably be humans first, animals second, rather than the opposite. For various reasons, the development of daptomycin and ramoplanin has been suspended for several years. If, during this period, these agents had been used as growth promoters, they would not now be under development for humans. I would rather see ramoplanin used for the microbial modulation of the intestinal tract in immunocompromised patients than as an animal-food additive.

During the last 30 years, thanks to molecular biology, enormous progress has been made in understanding the genetics and biochemistry of resistance. Incorporating this knowledge for decision-making in problems of public health importance is timely. I hope that it will not take 30 years for the pharmaceutical industry to act in agreement.

**Patrice Courvalin**
Institut Pasteur, Paris, France

### References

1. Courvalin P. Will avilamycin convert ziracine into zerocine? Emerg Infect Dis 2000;6:558.
2. Swann MM. Report of the Joint Committee on the use of antibiotics in animal husbandry and veterinary medicine. London: Her Majesty's Stationery Office; 1969.
3. Price KE, Godfrey JC, Kawaguchi H. Effect of structural modifications on the biological properties of aminoglycoside antibiotics containing 2-deoxystreptamine. In: Perlman D, editor. Structure-activity relationships among the semisynthetic antibiotics. New York: Academic Press; 1977. p. 272-4.
4. Chaslus-Dancla E, Martel JL, Carlier C, Lafont JL, Courvalin P. Emergence of 3-N-acetyltransferase IV in *Escherichia coli* and *Salmonella typhimurium* isolated from animals in France. Antimicrob Agents Chemother 1986;29:239-43.
5. Chaslus-Dancla E, Gerbaud G, Lafont JP, Martel JL, Courvalin P. Nucleic acid hybridization with a probe specific for 3-aminoglycoside acetyltransferase type IV: a survey of resistance to apramycin and gentamicin in animal strains of *Escherichia coli*. FEMS Microbiol Lett 1986;34:265-8.
6. Chaslus-Dancla E, Pohl P, Meurisse M, Marin M, Lafont JL. High genetic homology between plasmids of human and animal origins conferring resistance to the aminoglycosides gentamicin and apramycin. Antimicrob Agents Chemother 1991;35:590-3.
7. Fernandez-Munoz R, Monro RE, Torres-Pinedo R, Vasquez D. Substrate- and antibiotic-binding sites at the peptidyl-transferase centre of *Escherichia coli* ribosomes. Studies on the chloramphenicol, lincomycin and erythromycin sites. Eur J Biochem 1971;23:185-93.
8. Nikaido H. Multidrug efflux pumps of gram-negative bacteria. J Bacteriol 1996;178:5853-9.
9. Paulsen IT, Brown MH, Skurray RA. Proton-dependent multidrug efflux systems. FEMS Microbiol Rev 1996;60:575-608.
10. Rowe-Magnus DA, Mazel D. Resistance gene capture. Curr Opin Microbiol 1999;2:483-8.
11. Hall RM. Mobile gene cassettes and integrons: moving antibiotic resistance genes in gram-negative bacteria. Ciba Foundation Symposium 1997;207:192-202.
12. Hall RM, Stokes HW. Integrons: novel DNA elements which capture genes by site-specific recombination. Genetica 1993;90:115-32.
13. Nesvera J, Hochmannova J, Patek M. An integron of class 1 is present on the plasmid pCG4 from gram-positive bacterium *Corynebacterium glutamicum*. FEMS Microbiol Letters 1998;169:391-5.

